# Dynamics of Bose–Einstein Condensates Subject to the Pöschl–Teller Potential through Numerical and Variational Solutions of the Gross–Pitaevskii Equation

**DOI:** 10.3390/ma13102236

**Published:** 2020-05-13

**Authors:** Lucas Carvalho Pereira, Valter Aragão do Nascimento

**Affiliations:** 1 Programa de Pós-Graduaçāo em Ciência dos Materiais, Instituto de Física, Universidade Federal de Mato Grosso do Sul, Campo Grande 79070-900, Mato Grosso do Sul, Brazil; 2 Group of Spectroscopy and Bioinformatics Applied to Biodiversity and Health, School of Medicine, Postgraduation Program in Health and Development in the Midwest Region, Faculty of Medicine, Federal University of Mato Grosso do Sul, Campo Grande 79070-900, Mato Grosso do Sul, Brazil; aragao60@hotmail.com

**Keywords:** Bose–Einstein condensates, Pöschl–Teller potential, variational method, split-step Crank–Nicolson method

## Abstract

We present for the first time an approach about Bose–Einstein condensates made up of atoms with attractive interatomic interactions confined to the Pöschl–Teller hyperbolic potential. In this paper, we consider a Bose–Einstein condensate confined in a cigar-shaped, and it was modeled by the mean field equation known as the Gross–Pitaevskii equation. An analytical (variational method) and numerical (two-step Crank–Nicolson) approach is proposed to study the proposed model of interatomic interaction. The solutions of the one-dimensional Gross–Pitaevskii equation obtained in this paper confirmed, from a theoretical point of view, the possibility of the Pöschl–Teller potential to confine Bose–Einstein condensates. The chemical potential as a function of the depth of the Pöschl–Teller potential showed a behavior very similar to the cases of Bose–Einstein condensates and superfluid Fermi gases in optical lattices and optical superlattices. The results presented in this paper can open the way for several applications in atomic and molecular physics, solid state physics, condensed matter physics, and material sciences.

## 1. Introduction

The advent of Bose–Einstein Condensation opened the way for a better understanding of ultracold atoms trapped [1,2]. The dynamic and static properties of Bose–Einstein condensates (BEC) in the ultracold temperature regime are well described by the mean field equation known as the Gross–Pitaevskii equation (GPE) [3]. The manipulation of GPE has generated several applications in many areas of physics such as condensed-matter physics [4], nonlinear optics [5], optical solitons in nonlinear media (optical fibers) [6], metamaterials [7,8], etc.

The first BECs were produced using magnetic traps which are described by parabolic potentials [9,10]. However, there are other types of traps that are used in the confinement of ultracold atoms. Among the traps used in BECs, we can mention the optical traps known as optical lattices: periodic potentials created through the interference between lasers [4]. Since the optical lattices configurations are similar to the geometric arrangements of atoms in crystalline solids, they can be used to study atomic behavior in a highly controlled environment [11,12,13,14]. Thus, the ultracold atoms present in the optical lattice become a perfect environment for the investigation of nonlinear phenomena and solid-state physics [15,16]. There is also the possibility that magnetic and/or optical traps can be combined experimentally together or with other potentials. An important example of such a combination is the double-well potential [17,18].

Although there are many papers on confinement of ultracold atoms through the trap potentials mentioned above, it is interesting, from a theoretical and experimental point of view, to explore other atomic confinement potentials. To date, there is little research that considers the Pöschl–Teller potential (PT) as a potential for trapping cold atoms.

The PT potential due to its interesting physical properties has been studied in several situations. Jia et al. modeled a diatomic molecule using the PT potential from the experimental Rydberg–Klein–Rees (RKR) potentials for six diatomic molecules and obtained accurate results in fitting experimental RKR potential curves over a large range of internuclear distances for six molecules examined [19]. In addition, Yahia et al. obtained thermodynamic properties such as vibrational mean energy, vibrational specific heat, vibrational mean free energy and vibrational entropy for the PT-type potential for the Schrödinger and Klein–Gordon equations using the parametric Nikiforov–Uvarov method [20]. According to Park [21], two-dimensional transport of quasiparticles in bilayer graphene were also studied through a PT potential. The PT potential is used to account for the physical properties of many systems which includes the excitons, quantum wires, and quantum dots [22]. A very interesting property of this potential is the absence of reflection [23,24]—from the perspective of materials science, it can have formidable applications such as waveguides, solitons propagation in nonlinear media, complex systems, etc.

In recent years, some studies have shown significant results about BECs subject to the PT potential. In Ref. [25], a BEC of noninteracting ${}^{39}K$ atoms in this trapping potential, vortices in low-density neutron matter, and cold Fermi gases in Ref. [26], theoretical studies on cigar-shaped BEC in Ref. [27], and also, within cosmology, black holes as a gravitons condensate in Ref. [28]. However, from a general point of view, we can conclude that there are still few studies that explore the dynamics of BECs trapped in this potential. Thus, from the PT potential, new phenomena can be studied and understood in the BECs.

Motivated by the properties mentioned above and other properties that will be mentioned in Section 2.3, we propose in this paper to obtain variational and numerical solutions of EGP for a one-dimensionally confined BEC in a PT potential with attractive interactions. For the variational approximation (VA), we will use a Gaussian ansatz to minimize the effective Lagrangian of the proposed model [29]. For the numerical approximation, we will consider algorithms involving real- and imaginary-time propagation based on a split-step Crank–Nicolson (SSCN) method [30].

The paper is structured as follows: In Section 2, we formulate the dynamical model for our system and present how to obtain a one-dimensional GPE through a transversal parabolic confinement (harmonic potential) and a longitudinal hyperbolic confinement (Pöschl–Teller potential). In Section 3, we present the variational and numerical results obtained for the 1D-GPE. In Section 4, we discuss the results obtained and compare them with analytical results. In addition, finally, in Section 5, we present the conclusions and final considerations about this paper.

## 2. The Dynamical Model

### 2.1. The Gross–Pitaevskii Equation

The static and dynamical properties of a pure BEC made of *N* dilute and ultracold atoms are described by the three-dimensional Gross–Pitaevskii equation (3D-GPE) as follows:

(1) $$i\hbar \frac{\partial \Psi }{\partial t}=\left[-\frac{\hbar^{2}}{2m}\nabla^{2}+V_{ext}+g_{3D}{\left|\Psi \right|}^{2}\right]\Psi ,$$

where $i=\sqrt{-1}$ is the imaginary unit, ℏ=h/2π is the reduced Planck constant, *m* is the mass of each atom. The operator $\nabla^{2}$ is the Laplacian operator in three-dimensional cartesian coordinates given by $\nabla^{2}\equiv \partial^{2}/\partial x^{2}+\partial^{2}/\partial y^{2}+\partial^{2}/\partial z^{2}$, the term $V_{ext}$ is the external trapping potential and the nonlinearity coefficient,

(2) $$g_{3D}=\frac{4\pi \hbar^{2}a_{s}}{m},$$

is the strength of interatomic interaction, with $a_{s}$ the s-wave scattering length of atoms in the condensate; it is positive for repulsive interactions and negative for attractive interactions. In these equations, the quantity $\Psi \equiv \Psi \left(x,y,z,t\right)$ is the wave function of the BEC (also called the order parameter or macroscopic wave function) and, since ${\left|\Psi \right|}^{2}$ is defined as the atomic density of the *N* condensed atoms [31], Equation (1) will be supplemented by the normalization condition

(3) $$\int_{-\infty }^{+\infty }{\left|\Psi \right|}^{2}d^{3}r=N.$$

### 2.2. The External Potential

The external potential is responsible for BEC confinement. As mentioned in Section 1, there are several ways to confine ultracold atoms. In the case of the “standard” magnetic trap (MT), the external potential assumes the typical harmonic form

(4) $$V_{ext}^{MT}\left(x,y,z\right)=\frac{1}{2}m\left(\omega_{x}^{2}x^{2}+\omega_{y}^{2}y^{2}+\omega_{z}^{2}z^{2}\right),$$

where $\omega_{x}$, $\omega_{y}$, and $\omega_{z}$ are the trapping frequencies in the three different directions. The geometry of the trap and, hence, the shape of the condensate, may range from isotropic forms, to strongly anisotropic ones [32].

In this paper, we consider a BEC confined in a strongly elongated trap known as a “cigar-shaped” trap. In this configuration, which is highly anisotropic, the longitudinal and transverse confining frequencies (denoted by $\omega_{∥}$ and $\omega_{⊥}$, respectively) are such that $\omega_{∥}\ll \omega_{⊥}$, where $\omega_{∥}\equiv \omega_{x}$ and $\omega_{⊥}\equiv \omega_{y}=\omega_{z}$. This trap setting is interesting because the transverse confinement of the BEC is very narrow and causes the significant dynamics of cigar-shaped BEC to only occur in the longitudinal direction and thus the system effectively becomes one-dimensional. [31,32]. Thus, the external potential, $V_{ext}$, can be interpreted as a combination of a longitudinal, $V_{∥}$, and a transverse potential, $V_{⊥}$, that is,

(5) $$V_{ext}\left(x,y,z\right)=V_{∥}\left(x\right)+V_{⊥}\left(y,z\right).$$

In this paper, our objective is to reduce the 3D-GPE to one-dimensional Gross–Pitaevskii equation (1D-GPE) form, assuming, as mentioned above, that the BEC is held in a relatively tight cigar-shaped that correspond to the following transverse potential:

$$V_{⊥}\left(y,z\right)=\frac{1}{2}m\omega_{⊥}^{2}\left(y^{2}+z^{2}\right).$$

Based on these assumptions, we may decompose the wave function of the cigar-shaped BEC, Ψ, in a longitudinal (along *x*) and a transverse (on the y−z plane) component, and the system can be governed by 1D-GPE by assuming separable solutions on the form

(6) $$\Psi \left(x,y,z,t\right)=\psi \left(x,t\right)\Phi \left(y,z\right),$$

where the wave function Φ is well described by a solution of the transverse quantum harmonic-oscillator problem,

(7) $$\left[-\frac{\hbar^{2}}{2m}\nabla_{⊥}^{2}+\frac{1}{2}m\omega_{⊥}^{2}\left(y^{2}+z^{2}\right)\right]\Phi =E_{⊥}\Phi ,$$

where $\nabla_{⊥}^{2}\equiv \partial^{2}/\partial y^{2}+\partial^{2}/\partial z^{2}$ is the transverse Laplacian operator, and $E_{⊥}$ is the energy associated with transverse confinement of the trap. Since the considered system is effectively 1D, it is natural to assume that the transverse component of the condensate wavefunction, Φ, remains in the ground state [32]; Therefore, Φ takes the form

(8) $$\Phi \left(y,z\right)=\sqrt{\frac{m\omega_{⊥}}{\pi \hbar }}\exp\left[-\frac{m\omega_{⊥}}{2\hbar }\left(y^{2}+z^{2}\right)\right],$$

with $E_{⊥}=\hbar \omega_{⊥}$ being the ground state energy corresponding to transverse confinement.

Substituting Equation (8) into Equation (1) and integrating it over the transverse coordinate, we derive an effective 1D-GPE

$$i\hbar \frac{\partial \psi }{\partial t}=\left[-\frac{\hbar^{2}}{2m}\frac{d^{2}}{dx^{2}}+V_{∥}+\gamma g_{1D}{\left|\psi \right|}^{2}+\hbar \omega_{⊥}\right]\psi ,$$

with $g_{1D}=g_{3D}/2\pi a_{⊥}^{2}$ being the strength of one-dimensional interatomic interaction, $a_{⊥}=\sqrt{\hbar /\left(m\omega_{⊥}\right)}$ is the transversal harmonic oscillator ground state width and the coefficient $\gamma =sgn\left(a_{s}\right)=\pm 1$ characterizes the type of the s-wave interactions. In this paper, we propose a BEC with attractive interatomic interactions, i.e., γ=−1. It is found in the literature that the term $\hbar \omega_{⊥}$ is sometimes omitted because it does not affect the dynamics of the condensate [33].

### 2.3. The Pöschl–Teller Potential

The Pöschl–Teller potential [34] (here, the longitudinal potential $V_{∥}$) is a hyperbolic potential described mathematically by

(9) $$V_{∥}\left(x\right)=-\frac{\hbar^{2}V_{0}}{m\sigma^{2}}{\mathrm{sech}}^{2}\left(\frac{x}{\sigma }\right),$$

where $V_{0}$ and σ describe the amplitude/depth and width of this potential, respectively. The number of bound states of this potential is determined by a parameter *l* characterizing the depth of the potential energy [35]. This occurs when we rewrite the amplitude as $V_{0}\equiv l\left(l+1\right)$ and, as a consequence, by means of algebraic manipulations, we can reduce the one-dimensional Schrödinger equation subject to the PT potential to the Legendre differential equation [36,37]. Thus, the 1D-GPE for our model is given by

(10) $$i\hbar \frac{\partial \psi }{\partial t}=\left[-\frac{\hbar^{2}}{2m}\frac{d^{2}}{dx^{2}}-\frac{\hbar^{2}V_{0}}{m\sigma^{2}}{\mathrm{sech}}^{2}\left(\frac{x}{\sigma }\right)-g_{1D}{\left|\psi \right|}^{2}\right]\psi .$$

Finally, we can rewrite Equation (10) through the dimensionless variables:

(11) $$\psi \equiv \sqrt{\frac{N}{\sigma }}\tilde{\psi },\;x\equiv \sigma \tilde{x},\;t\equiv \frac{m\sigma^{2}}{\hbar }\tilde{t},\;g\equiv \frac{2\sigma a_{s}}{a_{⊥}^{2}}N$$

Therefore, Equation (10) takes the following rescaled form:

(12) $$i\frac{\partial \psi }{\partial t}=\left[-\frac{1}{2}\frac{d^{2}}{dx^{2}}-V_{0}{\mathrm{sech}}^{2}\left(x\right)-g{\left|\psi \right|}^{2}\right]\psi$$

with the rescaled 1D wave function subject to normalization

(13) $$\int_{-\infty }^{+\infty }{\left|\psi \right|}^{2}dx=1.$$

## 3. Results

The main equation in our model (Equation (12)) that describes a BEC trapped by the potential PT in a regime with attractive interactions can be rewritten as

(14) $$i\dot{\psi }+\frac{1}{2}\psi^{\prime \prime }+V_{0}{\mathrm{sech}}^{2}\left(x\right)\psi +g{\left|\psi \right|}^{2}\psi =0,$$

where $\psi^{\prime }\equiv \partial \psi /\partial x$ and $\dot{\psi }\equiv \partial \psi /\partial t$.

Provided the external potential is time independent, $V_{ext}\equiv V_{ext}\left(x,y,z\right)$, stationary solutions of the Equation (14) can be determined by expressing the condensate wave function as

(15) $$\psi \left(x,t\right)=\phi \left(x\right)e^{-i\mu t},$$

where ϕ is a real function and μ is the chemical potential which can be obtained from μ=∂E/∂N. Substitution of the Equation (15) into Equation (14) yields the following steady-state equation for ϕ:

(16) $$-\frac{1}{2}\phi^{\prime \prime }-V_{0}{\mathrm{sech}}^{2}\left(x\right)\phi -g{\left|\psi \right|}^{2}\phi =\mu \phi .$$

### 3.1. Variational Results

From the point of view of the variational method, Equation (14), together with normalization condition (Equation (13)), can be derived from the following Lagrangian density

$$L=\frac{1}{2}\phi^{\prime 2}-V_{0}{\mathrm{sech}}^{2}\left(x\right)\phi^{2}-\frac{g}{2}\phi^{4}-\mu \phi^{2}.$$

The variational solutions for Equation (14) were obtained by assuming an ansatz Gaussian form

(17) $$\phi \left(x\right)=\sqrt{\frac{M}{\pi^{1/2}w}}\exp\left(-\frac{x^{2}}{2w^{2}}\right),$$

where the variational parameters of soliton are the norm *M* and width *w*. It is important to note that the norma of the wave function, $M=\int_{-\infty }^{+\infty }\phi^{2}dx$, represents a conserved quantity.

The effective Lagrangian, *L*, is given by the integral

(18) $$L=\int_{-\infty }^{+\infty }Ldx+\mu ,$$

where the chemical potential, μ, was introduced to ensure that parameter *M* maintains the correct normalization of the wave function [15,16]. Substituting Equation (29) in Equation (18), we get

(19) $$L=\int_{-\infty }^{+\infty }\left[\frac{1}{2}\phi^{\prime 2}-V_{0}{\mathrm{sech}}^{2}\left(x\right)\phi^{2}-\frac{g}{2}\phi^{4}-\mu \phi^{2}\right]dx+\mu ,$$

and the substitution of ansatz given by Equation (17) in above effective Lagrangian yields:

(20) $$L=\mu \left(1-M\right)+\frac{M}{2w^{2}}-\frac{gM^{2}}{2\sqrt{2\pi }w}-\frac{MV_{0}}{\sqrt{\pi }}\int_{-\infty }^{+\infty }\frac{1}{w}{\mathrm{sech}}^{2}\left(x\right)e^{-x^{2}/w^{2}}dx.$$

The evolution equations for variational parameters can be obtained from the Euler–Lagrange equation:

(21) $$\frac{\partial L}{\partial q}=0$$

where *q* are the generalized coordinates $q\equiv \left\{\mu ,w,M\right\}$.

The first variational equation, ∂L/∂μ=0, recovers the normalization adopted in Equation (13), that is, M=1; which is substituted in the final forms of other equations below, except for equation ∂L/∂M=0, where M=1 is substituted after the differentiation. The other equations, ∂L/∂w=0 and ∂L/∂M=0, yield a set of coupled nonlinear integral equations which relates *w* and μ:

(22) $$\begin{array}{cc} 1= & \frac{gw}{2\sqrt{2\pi }}-\frac{V_{0}}{\sqrt{\pi }}\int_{-\infty }^{+\infty }\left(\frac{2x^{2}}{w}-w\right){\mathrm{sech}}^{2}\left(x\right)e^{-x^{2}/w^{2}}dx; \end{array}$$

(23) $$\begin{array}{cc} \mu = & \frac{1}{2w^{2}}-\frac{g}{\sqrt{2\pi }w}-\frac{V_{0}}{\sqrt{\pi }w}\int_{-\infty }^{+\infty }{\mathrm{sech}}^{2}\left(x\right)e^{-x^{2}/w^{2}}dx. \end{array}$$

### 3.2. Numerical Results

The numerical solutions of the Equation (14) were obtained through the Split-Step Crank–Nicolson (SSCN) method, using imaginary time propagation in order to obtain the BEC stationary ground state wave function. This method is well explained in Refs. [30,38]. The dimensioneless 1D-GPE, given by Equation (14), can be rewritten as

(24) $$i\frac{\partial \psi }{\partial t}={\hat{H}}_{GP}\psi .$$

The Hamiltonian, ${\hat{H}}_{GP}$, can be expressed by ${\hat{H}}_{GP}=\hat{T}+\hat{V}$, with $\hat{T}\equiv -\frac{1}{2}\frac{\partial^{2}}{\partial x^{2}}$ the kinetic energy operator and $\hat{V}\equiv {\hat{V}}_{ext}+{\hat{V}}_{NL}$ the potential energy operator with ${\hat{V}}_{ext}=-V_{0}{\mathrm{sech}}^{2}\left(x\right)$ and ${\hat{V}}_{NL}=-g{\left|\psi \right|}^{2}$. The term ${\hat{V}}_{NL}$ represents the nonlinear EGP term which will be treated as a potential. The solution of Equation (24) is

(25) $$\psi \left(x,t\right)=e^{-i{\hat{H}}_{GP}\Delta t}\psi \left(x,t_{0}\right),$$

where $\Delta t=t-t_{0}$. The operator $e^{-i{\hat{H}}_{GP}\Delta t}$ can be approximated via the Baker–Campbell–Hausdorff formula [39]. Thus, Equation (25) becomes

(26) $$\psi \left(x,t\right)=e^{-i\frac{\hat{V}}{2}\Delta t}e^{-i\hat{T}\Delta t}e^{-i\frac{\hat{V}}{2}\Delta t}\psi \left(x,t_{0}\right).$$

To apply the SSCN method, first, Equation (24) is discretized in the domain a<x<b using a spatial step Δx and a temporal step Δt. Then, we assume the initial condition, ψ(x,0), and the boundary conditions ψ(a,t) and ψ(b,t). For the stationary ground state, the wave function is essentially real and the method of propagating imaginary time (relaxation method) that deals with real variables seems to be convenient. The basic idea of the propagation of imaginary time is to replace the EGP temporal variable, *t*, with the imaginary temporal variable −it.

The temporal evolution of Equation (24) can be written in terms of the eigenfunctions $\phi_{n}$ with their respective eigenvalues $\mu_{n}$

(27) $$\psi \left(x,t\right)=\sum_{n=0}^{\infty }c_{n}\phi_{n}\left(x\right)e^{-i\mu_{n}t}.$$

The propagation of Equation (24) in imaginary time alters the temporal evolution given by Equation (27) to

(28) $$\psi \left(x,-it\right)=\sum_{n=0}^{\infty }c_{n}\phi_{n}\left(x\right)e^{-\mu_{n}t}.$$

Thus, all eigenfunctions will decay exponentially over time. However, all states excited will decay exponentially faster compared to the ground state. Consequently, only the ground state survives. That is, this procedure will converge to the lowest energy ground state solution [40]:

(29) $$\psi \left(x,t\right)\overset{-it}{⟶}\phi_{0}\left(x\right).$$

The real-time propagation of the SSCN method preserves the normalization of the wave function, while the propagation of the imaginary time of the SSCN method does not preserve normalization. This problem can be solved by restoring the normalization of the wave function after each step is performed. Thus, the method of propagating imaginary time for stationary problems in the ground state produces very accurate results at low computational cost.

In this paper, we use normalized Gaussian

(30) $$\psi \left(x,0\right)=\frac{1}{\sqrt{\pi^{1/2}w_{0}}}\exp\left(-\frac{x^{2}}{2w_{0}^{2}}\right),$$

as the initial condition and the Dirichlet boundary conditions to represent $\underset{x\to \pm \infty }{lim}\psi \left(x,t\right)=0$.

## 4. Discussion

**Figure 1:**
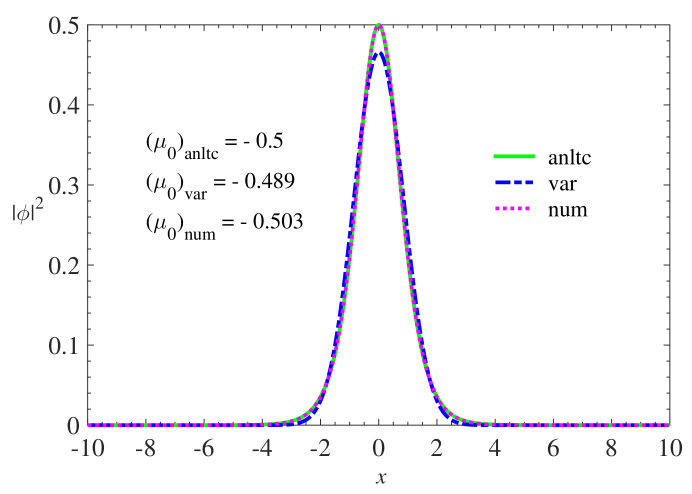
Comparison of variational and numerical results with the analytical solution of Equation (16) for $V_{0}=1/2$ and g=1. The analytical solution, represented by the continuous curve (green), is given by the expression $\phi \left(x\right)=\left(1/\sqrt{2}\right)\mathrm{sech}\left(x\right)$. The variational solution, represented by the dashed curve (blue), was obtained using the Gaussian ansatz $\phi \left(x\right)=1/\sqrt{\pi^{1/2}w}\exp\left(-0.5x^{2}/w^{2}\right)$ whose width, *w*, obtained by Equation (14) was w=1.211. The numerical solution, represented by the dotted curve (magenta), was obtained through the propagation of imaginary time using the Gaussian function $1/\sqrt{\pi^{1/2}w_{0}}\exp\left(-0.5x^{2}/w_{0}^{2}\right)$ as an initial condition. Here, labels “anltc”, “var”, and “num” represent analytical, variational, and numerical results, respectively.

**Figure 2:**
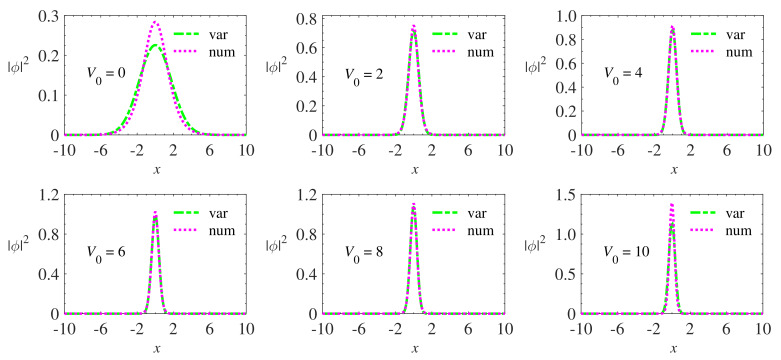
Variational and numerical results of the Equation (16) for different values of the Pöschl–Teller potential amplitude. It is noticed that, as the depth of the potential increases, the wave function width decreases and its amplitude increases, preserving the normalization. The nonlinearity coefficient remained fixed (g=1) in all simulations.

In particular, in the case $V_{0}=1/2$, for which results are presented below in Figure 1, Equation (16) with g=1 predicts the analytical solution $\phi \left(x\right)=\left(1/\sqrt{2}\right)\mathrm{sech}\left(x\right)$ whose corresponding chemical potential is μ=−0.5. Both variational and numerical results proved to be accurate in obtaining the solution of Equations (14) and (16) when compared with the analytical solution. These results are important from the point of view of the precision analysis of the variational and numerical method because the analytical solution becomes a “calibration” parameter for both methods. Variational and numerical results were also obtained for other values of the PT potential depth. Figure 2 shows the wave functions obtained in the simulations.

The results shown in Figure 2 indicate that the widths of the wave functions decrease and their amplitudes increase as the potential depth increases.

**Figure 3:**
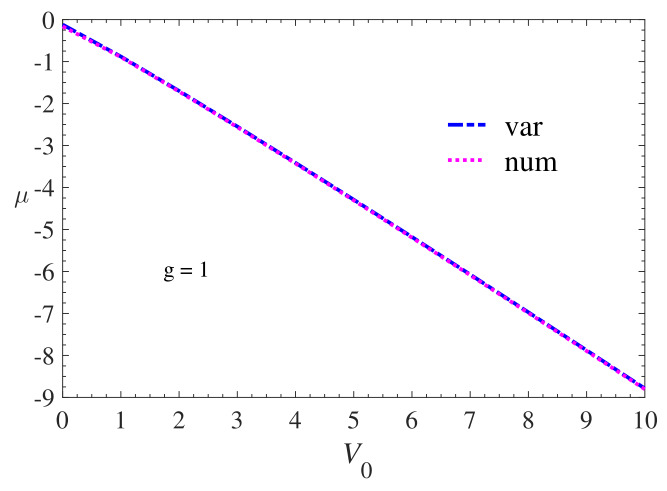
Variational and numerical results of chemical potential, μ, versus amplitude, $V_{0}$, of PT potential $-V_{0}{\mathrm{sech}}^{2}\left(x\right)$. We consider in this simulation g=1. The variational results are represented by the dashed curve (blue) while the numerical results are represented by the dotted curve (magenta). Similar behavior was observed in studies of stable gap solitons in SFGs trapped by optical lattices and optical superllatices.

The results for the values of the chemical potentials obtained by varying the amplitude of the potential can be interpreted through Figure 3. The values adopted for the amplitude are within the range $0\leq V_{0}\leq 10$. We can see that, as the depth of the potential increases, the chemical potential decreases through linear behavior. In this adopted interval, the results obtained from the chemical potential μ versus amplitude $V_{0}$ showed similar results observed in studies of Bose–Einstein condensates and Superfluid Fermi gases in optical lattices and optical superlattices (doubly periodic and quasiperiodic) [41,42,43,44,45].

## 5. Conclusions

In this paper, we consider the scenario where a BEC made up of diluted ultracold atoms with attractive interatomic interactions is transversally confined by a harmonic potential and longitudinally by the PT potential, assuming a cigar-shaped configuration. The results obtained demonstrated that the PT potential was able to trap BEC. The solutions showed stability and confirmed a behavior similar to the bright gap-solitons obtained in BECs and SFGs trapped by optical lattices. These results may open the way for a better understanding of ultracold quantum gases trapped by the PT potential and motivate new studies that may have applications in other areas of physics such as solid state physics, condensed matter physics, and materials sciences.
